# 1663. *Clostridioides difficile* testing following 18706 Pediatric Procedures: Implications for Diagnostic Stewardship

**DOI:** 10.1093/ofid/ofad500.1496

**Published:** 2023-11-27

**Authors:** Ruchita Negi, Abhishek Deshpande, Venkatraman Arakoni, Charles B Foster

**Affiliations:** Cleveland Clinic Children's, Strongsville, Ohio; Cleveland Clinic, Cleveland Clinic, Ohio; Cleveland Clinic, Cleveland Clinic, Ohio; Cleveland Clinic Children's, Strongsville, Ohio

## Abstract

**Background:**

*Clostridioides difficile* infection (CDI) poses a risk to surgical patients and antibiotic exposure may contribute to non-infectious diarrhea and unnecessary *C diff* testing (CDT). Our study evaluates the burden of CDT and CDI in children undergoing procedures with preoperative antibiotic prophylaxis.

**Methods:**

A retrospective cohort study (Jan 2013-Feb 2022) analyzed CDT by Toxin B PCR in children (2-18 years) undergoing sedated procedures with preoperative antibiotic prophylaxis at Cleveland Clinic Children’s. Two step testing with EIA to confirm CDI began in Jun 2018. Perioperative data from an anesthesia database were joined with CPT4 codes and organized by procedure categories used for prophylactic antibiotic selection. CDT and CDI rates by PCR for postoperative days 3-30 were compared based on antibiotic choice (cefazolin as reference) and procedure type (orthopedics as reference) using rate ratios (RR) and mid-p exact test.

**Results:**

A total of 14229 children underwent 18706 procedures. Postoperative CDT, PCR negative and PCR positive rates were 12.2, 10.3 and 1.9/1000 procedures, respectively (Table 1). Of 11 PCR positive tests followed by EIA, 8 were positive (73%). CDT rates were highest for gastrointestinal (GI; 48.4), thoracic (20.8) and neurosurgery (12.8) procedures (Figure 1). CDI rates by PCR were increased for GI procedures (5.9; *P* < 0.001) and did not differ by antibiotic category. Compared to orthopedic procedures, the RR for negative CDT was highest for GI procedures 71.46 (28.73-229.4; *P < 0.001*). For antibiotics (Figure 2), the lowest CDT rate was for cefazolin (7.1). Compared to cefazolin, the negative CDT RR was 16.23 (10.97-23.69; *P* < 0.001) for metronidazole, 10.06 (7.23-13.95; *P* < 0.001) for broad spectrum antibiotics and 8.39 (5.60-12.36; *P* < 0.001) for vancomycin.

C difficile Negative and Positive Testing Rates in the 30-Days Following Pediatric Procedures Requiring Preoperative Antibiotic Prophylaxis, Stratified According to Prophylactic Antibiotic Category and Procedure Category Used to Select Prophylactic Antibiotic

**Figure:**
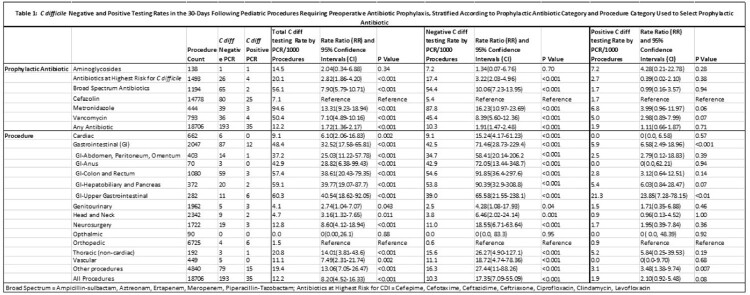

**Figure:**
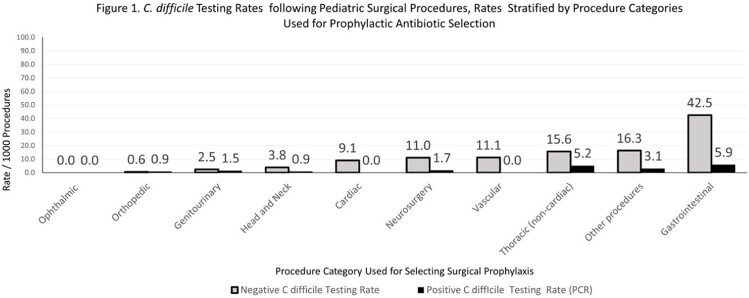

**Figure:**
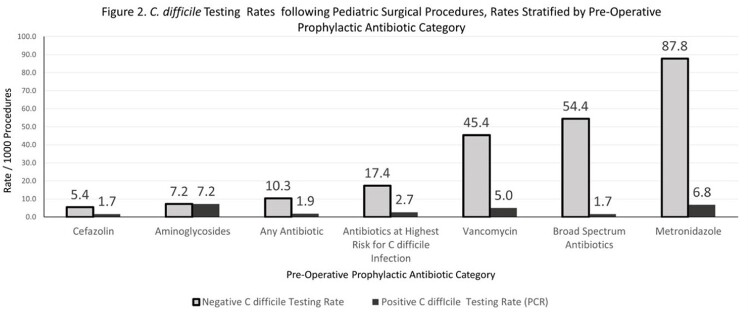

**Conclusion:**

The study demonstrates that CDT rates by PCR vary by procedure type and antibiotic prophylaxis regimen. Both CDI and CDT rates were higher for GI procedures. Negative CDT rates were especially high for GI procedures and for antibiotic prophylaxis with non-cefazolin containing regimens. High postoperative rates of negative CDT suggest that non-infectious diarrhea contributes to testing. These studies are relevant to diagnostic stewardship efforts aimed at preventing unnecessary CDT.

**Disclosures:**

**Abhishek Deshpande, MD; PhD**, Merck: Advisor/Consultant|Seres Therapeutics: Grant/Research Support **Charles B. Foster, MD**, 3M Co: Stocks/Bonds|Novavax: Stocks/Bonds|Scynexis: Stocks/Bonds|Veru Inc: Stocks/Bonds|Vir Biotechnology: Stocks/Bonds

